# Analysis of variants in *GATA4* and *FOG2*/*ZFPM2* demonstrates benign contribution to 46,XY disorders of sex development

**DOI:** 10.1002/mgg3.1095

**Published:** 2020-01-21

**Authors:** Jocelyn A. van den Bergen, Gorjana Robevska, Stefanie Eggers, Stefan Riedl, Sonia R. Grover, Philip B. Bergman, Chris Kimber, Ashish Jiwane, Sophy Khan, Csilla Krausz, Jamal Raza, Irum Atta, Susan R. Davis, Makato Ono, Vincent Harley, Sultana M. H. Faradz, Andrew H. Sinclair, Katie L. Ayers

**Affiliations:** ^1^ Genetics Murdoch Children's Research Institute Parkville Vic. Australia; ^2^ Research Genomics Murdoch Children's Research Institute Parkville Vic. Australia; ^3^ St. Anna Children's Hospital Medical University of Vienna Vienna Austria; ^4^ Paediatric Department Medical University of Vienna Vienna Austria; ^5^ Department of Paediatric and Adolescent Gynaecology Royal Children's Hospital Melbourne Parkville Vic. Australia; ^6^ Department of Paediatrics University of Melbourne Melbourne Vic. Australia; ^7^ Department of Paediatric Endocrinology and Diabetes Monash Children's Hospital Clayton Vic. Australia; ^8^ Department of Paediatrics Monash University Clayton Vic. Australia; ^9^ Department of Paediatric Urology Monash Children's Hospital Clayton Vic. Australia; ^10^ Department of Urology Sydney Children's Hospital Randwick Randwick NSW Australia; ^11^ Surgical Department Angkor Hospital for Children Siem Reap Cambodia; ^12^ Department of Experimental and Clinical Biomedical Sciences“Mario Serio” University of Florence Firenze Toscana Italy; ^13^ Paediatric Department National Institute of Child Health Karachi City Sindh Pakistan; ^14^ Women's Health Research Program School of Public Health and Preventive Medicine Monash University Melbourne Vic. Australia; ^15^ Department of Paediatrics Tokyo Bay Urayasu Ichikawa Iryo Center Urayasu Chiba Japan; ^16^ Centre for Endocrinology and Metabolism Hudson Institute of Medical Research Clayton Vic. Australia; ^17^ Division of Human Genetics Centre for Biomedical Research Faculty of Medicine Diponegoro University (FMDU) Semarang Indonesia

**Keywords:** disorders of sexual development, *FOG2*, functional analysis, *GATA4*, mutations, *ZFPM2*

## Abstract

**Background:**

GATA‐binding protein 4 (*GATA4*) and Friend of GATA 2 protein (*FOG2*, also known as *ZFPM2*) form a heterodimer complex that has been shown to influence transcription of genes in a number of developmental systems. Recent evidence has also shown these genes play a role in gonadal sexual differentiation in humans. Previously we identified four variants in *GATA4* and an unexpectedly large number of variants in *ZFPM2* in a cohort of individuals with 46,XY Differences/Disorders of Sex Development (DSD) (Eggers et al, Genome Biology, 2016; 17: 243).

**Method:**

Here, we review variant curation and test the functional activity of *GATA4* and *ZFPM2* variants. We assess variant transcriptional activity on gonadal specific promoters (*Sox9* and *AMH*) and variant protein–protein interactions.

**Results:**

Our findings support that the majority of *GATA4* and *ZFPM2* variants we identified are benign in their contribution to 46,XY DSD. Indeed, only one variant, in the conserved N‐terminal zinc finger of *GATA4*, was considered pathogenic, with functional analysis confirming differences in its ability to regulate *Sox9* and *AMH* and in protein interaction with *ZFPM2*.

**Conclusions:**

Our study helps define the genetic factors contributing to 46,XY DSD and suggests that the majority of variants we identified in *GATA4* and *ZFPM2/FOG2* are not causative.

## Graphical abstract

We identified a number of 46,XY DSD individuals with variants in *GATA4* and *FOG2*. Variant curation and functional analysis revealed the majority of these variants are not likely to be causative.

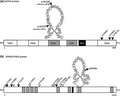

**Abbreviations**
AVSD: Atrial ventricular septal defects
CHD: congenital heart disease
DIH3: diaphragmatic hernia 3
DSD: differences/disorders of sex development
HEK293: human embryonic kidney 293
OMIM: online mendelian inheritance in man
SNV: single nucleotide variant
SRXY9: 46XY sex reversal 9
TAD: transactivation domain
TOF: tetralogy of fallot

## 1 INTRODUCTION

The GATA zinc finger transcription factors (1–6) are an evolutionally conserved family that plays various roles in embryonic development. The GATA members have two zinc finger domains that are required for recognition and affinity for DNA and interaction with other transcription factors (Morrisey, Ip, Tang, & Parmacek, 1997; Yang & Evans, 1992). The *GATA4* family member has been shown to be involved in development of the heart, pancreas, liver, foregut as well as in the genital ridge of both sexes (Jacobsen et al., 2002; Kuo et al., 1997; Molkentin, 2000; Molkentin, Lin, Duncan, & Olson, 1997; Ritz‐Laser et al., 2005; Schrade et al., 2015). In humans, variants in *GATA4* (MIM# 600576) were first identified in patients with congenital heart disease (CHD) including Atrial ventricular septal defects (AVSD) (Garg et al., 2003; Rajagopal et al., 2007) and Tetralogy of Fallot (TOF)(Zhang et al., 2008).

A number of cell‐specific co‐factors have been shown to interact with *GATA4* and influence its transcriptional activity. The essential multi zinc finger protein *FOG2* (*ZFPM2*; MIM# 603693) directly interacts with *GATA4*, by forming a heterodimer that represses expression of *GATA4* target genes (Svensson, Huggins, Dardik, Polk, & Leiden, 2000; Svensson, Tufts, Polk, & Leiden, 1999). In mice a knock‐in mutation of *Gata4* and a modified knock‐out of *Fog2/Zfpm2* both show testicular anomalies characterized by a failure to up‐regulate *Sry* and *Sox9* (Manuylov, Fujiwara, Adameyko, Poulat, & Tevosian, 2007; Manuylov et al., 2011; Tevosian et al., 2002). Although the molecular mechanisms are unclear, the direct interaction between *Gata4* and *Zfpm2* are thought to be required for differentiation of testis cell lineages (Bouma, Washburn, Albrecht, & Eicher, 2007), yet just a handful of reports have found *GATA4* and *ZFPM2* variants in humans with DSD. In 2011, a heterozygous missense variant in the *GATA4* gene was identified in a family with two affected brothers, one presented with ambiguous genitalia and inguinal hernia at birth, the other was diagnosed later in life to also have testicular anomalies (Lourenco et al., 2011). The variant was also present in the unaffected mother, however other female relatives and 46,XY affected individuals had heart anomalies (from systolic murmur to Tetralogy of Fallot). The variant c.661G > A (p.G221R) was located in the N‐terminal zinc finger domain and had reduced DNA binding and transcriptional activity, as well as reduced interaction with co‐factor protein *ZFPM2*/*FOG2* (Lourenco et al., 2011). A report of missense variants in *ZFPM2* in two probands was published in 2014 (Bashamboo et al., 2014). One proband (with a heterozygous variant; c.1206T > A [p.S402R]) presented with 46,XY complete gonadal dysgenesis, and evidence suggested familial inheritance. While the second proband was born with ambiguous genitalia and testicular tissue (46,XY partial gonadal dysgenesis) with two variants in the *ZFPM2* gene; a homozygous missense variant c.1631G > A (p.M544I) and a heterozygous change c.779G > A (p.R260Q) (Bashamboo et al., 2014).

While screening 279 46,XY DSD individuals for variants in genes known to cause DSD, we previously identified four variants in *GATA4* and 10 variants in *ZFPM2* in 16 patients (Eggers et al., 2016). This was a surprisingly large number of variants for *ZFPM2* considering only one paper had previously implicated this gene in DSD (Bashamboo et al., 2014). A large proportion of these variants were previously reported in association with congenital heart defects (CHD) but a lack of supporting evidence led us to classify many of these *GATA4* and *ZFPM2* variants as variants of unknown significance (VUS). Here we have re‐curated these *GATA4* and *ZFPM2* variants using updated tools, and have tested their molecular activity in the context of gonadal signaling using several in vitro assays.

## 2 RESULTS

### 2.1 *GATA4* variants identified in 46,XY DSD individuals

In our previous study (Eggers et al., 2016), we identified a number of affected individuals with heterozygous missense variants in *GATA4* (MIM# 600576) (four variants in seven patients), detailed in Table 1. Of the seven individuals with *GATA4* variants, five presented with hypospadias (case 2, 3, 5–7). Case 5 in addition to hypospadias presented with multiple congenital anomalies including imperforate anus and dysmorphic facial features (Table 1). While, case 4 presented as a nonvirilized female with inguinal testes and no uterus. No hormonal data were available to confirm androgen insufficiency; however, our panel did identify a previously described variant in Androgen Receptor (AR:NM_000044:exon7:c.2599G > A:p.Val867Met) (MIM# 313700) in association with androgen resistance syndrome (AIS; MIM# 300068), consistent with the patient's phenotype (detailed in Table 1 and Table S1). Case 1 had a familial history of micropenis and cryptorchidism with a similar phenotype also present in a maternal uncle. The c.684G > C (p.Trp228Cys) variant identified in the proband and uncle was also reported by LaPiscina (Martinez de LaPiscina et al., 2018). A large number of ClinVar reported variants in *GATA4* are associated with various forms of CHD, including three of the variants we identified c.1037C > T (p.Ala346Val), c.1180C > A (p.Pro394Thr), and c.1220C > A (p.Pro407Gln), in association with Atrio ventricular septal defect (AVSD4; MIM# 614430) (Table 1).

**Table 1:** *GATA4* and *ZFPM2/FOG2* variants identified in 46,XY DSD cohort

Patient Id	Karyotype	Ancestry	External genitalia	Gonads	Sex of rearing	Other DSD variant	cDNA: NM_002052.4	Protein: NP_002043	Zygosity	Inherit.	ClinVar: Condition	Variant class^a^
*Variants identified in GATA4*												
1	46,XY	EUR	Micropenis, cryptorchidism	Testis: Immature tubules, Sertoli‐only phenotype, spermatogonia absent, calcifications present	Male	N	c.684G > C	p.W228C	het	M(1) A(1)	n/a	P
2	46,XY	KHM	Perineal hypospadias, chordee and penoscrotal transposition	Bilateral descended testes	Male	N	c.1037C > T	p.A346V	het	n/a	AVSD4	LB
3^b^	46,XY	PAK	Perineal hypospadias	Unknown	Male	Y^b^	c.1180C > A	p.P394T	het	n/a	AVSD4	B
4^c^	46,XY	EUR	Female (no virilization)	Inguinal bilateral testis, no uterus	Female	Y^c^	c.1180C > A	p.P394T	het	n/a		B
5^d^	46,XY	IDN	Penile hypospadias, imperforate anus	Bilateral descended testes	Male	N	c.1220C > A	p.P407Q	het	n/a	TOF, AVSD4, ASD2, VSD1	B
6	46,XY	IDN	Scrotal hypospadias	Testes palpable, hypoplastic uterus	Male	N	c.1220C > A	p.P407Q	het	n/a		LB
7	46,XY	IDN	Perineal hypospadias	Bilateral inguinal testes	Male	N	c.1220C > A	p.P407Q	het	n/a		LB

Patient id	Karyotype	Ancestry	External genitalia	Gonads	Sex of rearing	Other DSD variant	cDNA NM_012082.3:	Protein NP_036214:	Zygosity	Inherit.	ClinVar: Condition	Variant class^a^
*Variants identified in ZFPM2/FOG2*												
8	46,XY	PAK	Perineal hypospadias	Unknown	Male	N	c.89A > G;	p.E30G;	het	n/a	SRXY9; TOF; DORV; DIH3	LB
							c.1612G > A	p.V538I	het	n/a	n/a	VUS
9	46,XY	EUR	Ambiguous (female after surgery)	Ovotesticular	Female	N	c.89A > G;	p.E30G;	het	n/a	SRXY9; TOF; DORV; DIH3	LB
							c.1255G > A	p.E419K	het	n/a	n/a	VUS
10	46,XY	IDN	Perineal hypospadias	Testis not found, indications of decreased gonadal function	Male	N	c.292G > A	p.D98N	het	n/a	SRXY9	B
11	46,XY	EUR	Female	Streak gonads, müllerian structures present	Female	N	c.292G > A	p.D98N	het	n/a		B
12	46,XY	EUR	Ambiguous	Ovotesticular	Female	N	c.629G > C	p.S210T	het	M(1); F(0)	SRXY9	B
3^b^	46,XY	PAK	Perineal hypospadias	Unknown	Male	Y^b^	c.1003C > G	p.L335V	het	n/a	n/a	B
13	46,XY	EUR	Female, blind vagina and rudimentary uterus	Gonadal agenesis	Female	N	c.1632G > A	p.M544I	het	n/a	SRXY9	LB
14	46,XY	PAK	Perineal hypospadias	Unknown	Male	N	c.1770G > C	p.K590N	het	n/a	n/a	VUS
15^c^	46,XY	KHM	Micropenis, penoscrotal hypospadias	Bilateral descended testes	Male	Y^c^	c.1818_1820del	p.L607del	het	M(0)	n/a	VUS
16^c^	46,XY	IDN	Clitoromegaly, one perineal opening	Inguinal, atrophic dysgenic testes, carcinoma in situ and seminoma	Female	Y^c^	c.2107A > C	p.M703L	het	n/a	DIH3; DORV	B

Note

Ancestry: EUR (European); KHM (Cambodian); PAK (Pakistan); IDN (Indonesian).

Inherit. (Inheritance): M (maternal), F (father), A (affected family member); (0) variant allele not present, (1) variant allele present; (n/a) data not available.

ClinVar annotation and associated OMIM phenotype: AVSD4 (Atrioventricular septal defect 4; MIM# 614430); TOF (Tetralogy of Fallot; MIM# 187500); VSD1 (Ventricular septal defect 1; MIM# 614429); ASD2 (Atrial septal defect 2; MIM# 607941); VSD1 (Ventricular septal defect 1; MIM# 614429); DORV (double outlet right ventricle); DIH3 (Diaphragmatic hernia 3; MIM# 610187); SRXY9 (46,XY Sex reversal 9; MIM# 616067); TOF (Tetralogy of Fallot; MIM# 187500); (n/a) variant not reported in ClinVar.

^a^ Variant class. (Variant classification): P (pathogenic), B (benign), LB (likely benign), VUS (variant of unknown significance), according to re‐curation, see Table S2.

^b^ Case 3 has a *GATA4* and *FOG2* missense variant.

^c^ Patients with variants in other DSD genes (see Table S1 for variant details).

^d^ Case5: multiple congenital anomalies noted (dysmorphic facial features, hypertelorism, web neck, low posterior hairline, clinodactyly of 4‐5th toes, not investigated for CHD).

**Figure 1:**
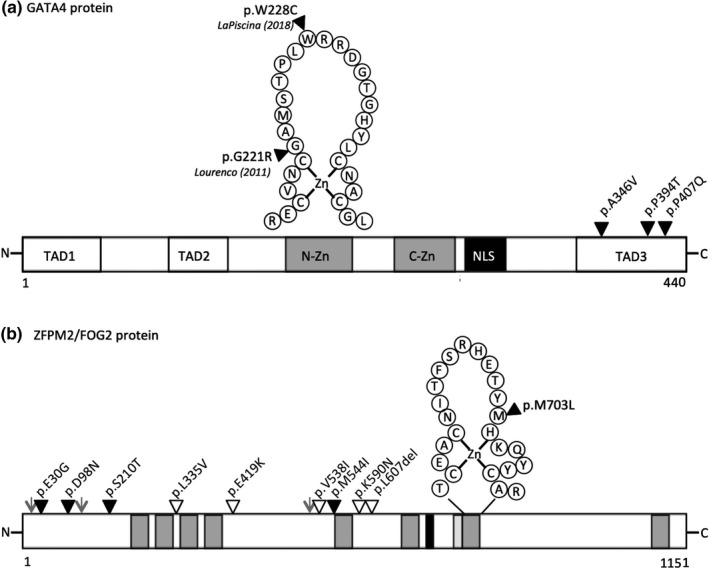
Protein schematic of *GATA4* and *ZFPM2*/*FOG2* showing coding variants identified in 46,XY individuals. (a) The human *GATA4* protein (NP_002043) is 440 amino acids with the following functional elements: two highly conserved N‐terminal and C‐terminal zinc finger domains (N‐Zn and C‐Zn, grey filled boxes); three transactivation domains (TAD1‐3); and a nuclear localization signal (NLS, black box). Positions of four *GATA4* heterozygous missense variants identified in four individuals are shown. One variant p.W228C (case1) initially identified by our study and later reported by LaPiscina et al. occurs in the N‐terminal zinc finger, along with the first variant published in association with 46,XY DSD by Lourenco et al. (used as a positive control in this study). The other three variants (p.A346V, p.P394T, p.P407Q) occur in the C‐terminal transactivation domain (TAD3) and are reported in ClinVar in association with CHD (solid black triangle). (b) The human *ZFPM2* protein (NP_036214) is 1,151 amino acids long with the following functional elements: eight zinc finger domains (grey filled boxes); nuclear localization signal (NLS) (solid black box); a CTBP2 interaction domain (light grey box); residues known to undergo post‐translational modifications (grey arrow head). Position of nine *ZFPM2* heterozygous missense and one in‐frame deletion variants found in ten individuals. All variants lie outside of known functional domains, except the p.M703L variant which occurs within the 7th zinc finger domain. The solid black triangles represent variants reported in ClinVar, the white triangles indicate unreported variants

The *GATA4* protein has two functionally conserved Zn‐finger domains that are essential for DNA binding and protein‐protein interactions. The variant we found in case 1 (p.W228C) was located in the N‐terminal Zn‐finger (Figure 1a). This amino acid position is highly conserved and in silico algorithms suggests the residue is not tolerant to substitutions (has the maximum Grantham score of 215; while in silico predictors consistently deemed the change to be damaging) (Table 1). The other three *GATA4* variants we identified (p.A346V, p.P394T, p.P407Q) were located in the C‐terminal TAD (Figure 1a).

### 2.2 *ZFPM2* variants identified in 46,XY DSD individuals

In our cohort, we identified 10 *ZFPM2* (MIM# 603693) variants in 10 patients occurring in a wide spectrum of 46,XY DSD phenotypes ranging from males with hypospadias (case 3, 8, 10, 14, 15), ambiguous genitalia (case 9, 12) to 46,XY individuals presenting with female external genitalia (case 11, 13, 16) (Table 1).

About half (5) of the *ZFPM2* variants we identified have not been previously reported, while the other five variants have been reported in ClinVar in association with congenital heart defects (TOF; MIM# 187500), diaphragmatic hernia (DIH3; MIM# 610187), or 46, XY DSD (SRXY9; MIM# 616067). Interestingly, two of the patients (case 8 and 9) each had a novel variant and an identical second hit in a more commonly observed variant (c.89A > G, p.Glu30Gly) that has been reported in ClinVar with numerous *ZFPM2*‐associated conditions. In these cases it is not known whether the variants effect the same or different alleles (Table 1).

We also mapped whether the *ZFPM2* amino acid changes occur in annotated functional domains. The *ZFPM2* (*FOG2*) protein has eight Zn‐finger domains, and an N‐terminal region (1–247) that is required for *GATA4*‐mediated repression of target genes (Svensson et al., 2000). Only one variant is located in an annotated domain, the 6th Zn finger c.1632G > A (p.Met703Leu); while four cluster around the 5th finger c.1612G > A (p.Val538Ile), c.1632G > A (p.Met544Ile), c.1770G > C (p.Lys590Asn), c.1818_1820del (p.Leu607del). The other five variants are within the N‐terminal region, c.89A > G (p.Glu30Gly), c.292G > A (p.Asp98Asn); c.629G > C (p.Ser210Thr), c.1255G > A (p.Glu419Lys) (Figure 1b).

### 2.3 Re‐curation of identified variants using updated filtering and curation guidelines

In our previous study, variant filtering and curation focused on the 64 known diagnostic DSD genes (Eggers et al., 2016). Variant curation was based on the following criteria: population database global minor allele frequency (MAF) (using ExAC), protein prediction tools and clinical variant databases (ClinVar, HGMD). Current tools and guidelines to filter and curate variants have evolved rapidly since our initial publication. Therefore, we have re‐curated the 5 *GATA4* and 10 *ZFPM2* variants using current population databases (GnomAD v2.1 (Karczewski et al., 2019)), prediction tools, and implemented a scoring criterion based on the American College of Medical Genetics and Genomics (ACMG) guidelines (Nykamp et al., 2017; Richards et al., 2015). For full details see Table S2 and materials and methods section.

Based on re‐curation, three *GATA4* missense variants (p.A346V, p.P394T, p.P407Q) were reclassified as likely benign or benign. Primarily, the global MAF or Popmax filtering AF (95% CI) in GnomAD was higher than the expected frequency of the condition (a threshold of 0.4%, based on the prevalence of hypospadias at 1/250 male births (Blaschko, Cunha & Baskin, 2012)). Protein function predictions were often inconsistent therefore providing benign supporting evidence. Due to limited clinical information and lack of segregation data, patient‐guided criteria contributed minimally to variant classification.

The *GATA4* variant identified in case 1 (p.W228C) was curated as pathogenic. The variant is present in a highly conserved domain without benign variation and absent from population databases. Additional pathogenic supporting evidence was confirmed by familial segregation of the variant in the affected maternal uncle and genotype–phenotype correlation (see Table S2).

Reclassification of the ten *ZFPM2* variants revealed six missense were considered benign or likely benign (p.E30G, p.D98N, p.S210T, p.L335V, p.M544I, p.M703L). While three missense (p.V538I, p.E419K, p.K590N) and one in frame deletion (p.L607del) were re‐classified as variants of unknown significance (VUS) due to conflicting or lack of evidence to support classification. Similarly, to individuals with *GATA4* variants, limited patient information was available for curation (Table S2).

### 2.4 Oligogenic inheritance in other DSD genes

Oligogenic inheritance of DSD variants was observed for four individuals with a *GATA4* or *ZFPM2* variant. Both probands with the *GATA4* p.P394T variant had an additional DSD gene variant; case 3 *ZFPM2*:NM_012082:c.1003C > G (p.L335V) (benign classification) and case 4 had a well‐described pathogenic variant in the ligand binding domain of the androgen receptor (AR:NM_000044.4:exon7:c.2599G > A (p.V867M)) (Abilash et al., 2016; Li et al., 2017; Lubahn et al., 1989) in association with androgen resistance syndrome (AIS; MIM# 300068), consistent with the patients phenotype (Table S1). Two individuals with a *ZFPM2* variant also had additional variants in diagnostic DSD such as *AR* and *NR5A1* which also correlate with the described 46,XY phenotype (case 15: AR:NM_000044.4:exon5:c.2191G > A (p.V731M) and case 16: *NR5A1*:NM_004959.4:c.251G > A (p.R84H) (Köhler et al., 2008; Robevska et al., 2017). In cases 4, 15, and 16, *GATA4* and *ZFPM2* variants were curated as benign supporting evidence but were included as potential risk factors that may contribute to the severity of the DSD phenotype (Table S1 for additional DSD variant description).

### 2.5 Transcriptional control of gonadal specific promoters by *GATA4* and *ZFPM2*

**Figure 2:**
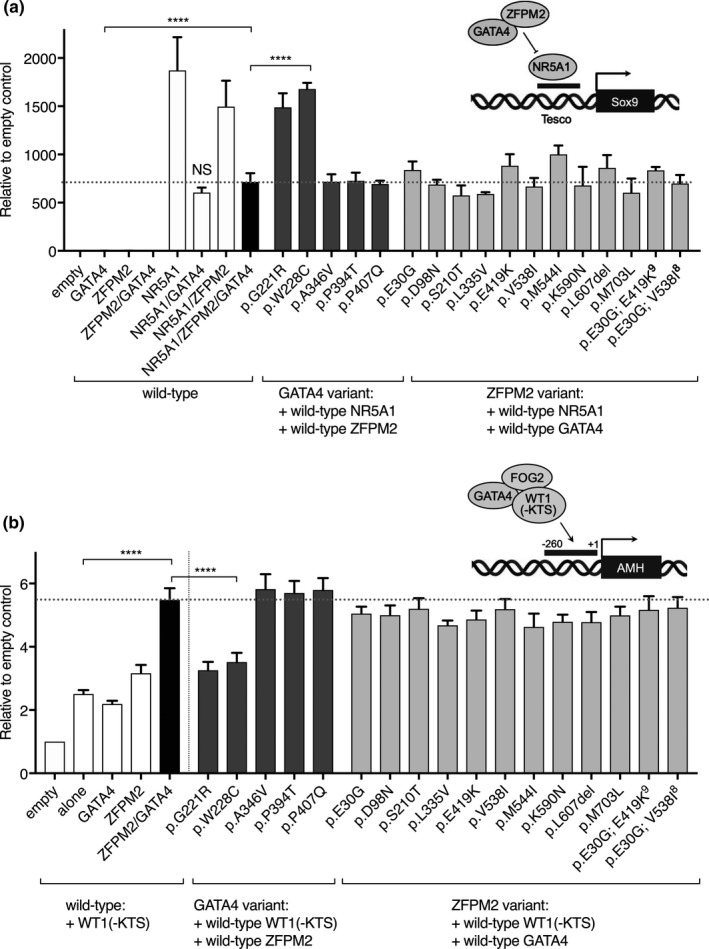
Transactivation assays assessing *GATA4*, *ZFPM2*/*FOG2* and co‐factor activity on gonadal promoter elements. (a) Transcription factors *GATA4*, *ZFPM2* and co‐factor *NR5A1* were transfected into HEK293 cells and transcriptional activity of mTesco enhancer element construct was measured with luciferase as a reporter. Wild‐type transcription factors were tested individually as well as in combination, empty vector was used as a negative control (white and black bars). Wild‐type co‐factor *NR5A1* and *ZFPM2* were transfected with individual *GATA4* variants (dark grey bars), or wild‐type *NR5A1* and *GATA4* with *ZFPM2* variants (light grey). Maximal transactivation of mTesco was observed with wild‐type *NR5A1* alone. Wild‐type *GATA4* or *ZFPM2* (alone or in combination) is able repress *NR5A1* activation. Most variants tested were able to maintain repression of *NR5A1* activation of mTesco (compared to wild‐type *NR5A1*/*ZFPM2*/*GATA4*, black bar, horizontal dotted line), only *GATA4* p.G221R (previously published positive control, Lourenco et al.) and p.W228C variants showed loss of *NR5A1* repression. (b) Transcriptional activity of *GATA4*, *ZFPM2* and co‐factor WT1 (−KTS) on human *AMH* promoter (+10–[−270]) assessed by luciferase assays. Wild‐type transcription factors were tested individually as well as in combination, empty vector was used as a negative control (white and black bars). Wild‐type co‐factor WT1 (−KTS) and *ZFPM2* were transfected with individual *GATA4* variants (dark grey bars), or wild‐type WT1 (−KTS) and *GATA4* with *ZFPM2* variants (light grey bars). Maximal transactivation of *hAMH* was observed with wild‐type WT1 (−KTS), *ZFPM2* and *GATA4* (black bar, horizontal dotted line). A similar level of transactivation compared to the wild‐type was observed for the majority of variants tested, except for *GATA4* p.G221R and p.W228C variants which showed loss of activation. For all transactivation assays: Data represented as the mean and *SEM* of at least three independent experiments (*n* = 3), as a fold change relative to the empty vector control (background), each assay was run in technical triplicate. P‐values were calculated using a one‐way ANOVA multiple comparisons (Dunnett test) (compared to the wild‐type—black bar, horizontal dashed line), *p*‐value **** < .0001

A large proportion of variants identified in *GATA4* and *ZFPM2* were classified as likely benign or benign under our new curation guidelines. Despite this, many of these variants were also reported in association with CHD but their role in DSD is unclear. To determine whether *GATA4* and *ZFPM2* variants specifically affect testis signaling we tested the ability of over‐expression constructs to activate gonadal promoters using dual luciferase reporter assays in HEK293 cells. Whilst *ZFPM2* and *GATA4* alone are not able to trans‐activate mTesco (the mouse enhancer of *Sox9*) (Bashamboo et al., 2014) (Figure 2a), *NR5A1* is a known activator (Sekido & Lovell‐Badge, 2008), and *GATA4* and *ZFPM2* can repress this activation. Indeed, when we co‐transfected *NR5A1* with *ZFPM2* alone we saw a 30% reduction, while with *GATA4* or *GATA4* + *ZFPM2*, we saw a 50% reduction (*p* < .0001) in *NR5A1* activation of mTesco (*p* < .0001) (Figure 2a). We then tested *GATA4* variants with *ZFPM2* wild‐type (or vice versa) using the previously described deleterious variant *GATA4* p.G221R, as a positive control (Lourenco et al., 2011) (Figure 2a). Both the control *GATA4* variant p.G221R and case 1 variant (p.W228C) with wild‐type *ZFPM2* had a loss of repression of *NR5A1* mediated activation of mTesco (comparable to *NR5A1*/*ZFPM2* wild‐type levels without *GATA4*) (*p* < .0001) (Figure 2a). The other variants in *GATA4* showed a very similar level of mTesco activity as the wild‐type. None of the 10 *ZFPM2* variants significantly affected repression, suggesting these variants are still able to form a complex with *GATA4* and repress *NR5A1* activity (Figure 2a). Two patients (case 8 and 9) had two heterozygous missense variants in *ZFPM2*; it is not known whether these variants are in cis‐ or trans‐. However, we tested them individually and in combination but no loss of activity was observed (Figure 2a).

Several publications have highlighted *AMH* as a target of *GATA4* activity (Tremblay, 1999; Tremblay & Viger, 2001, 2003; Viger, Mertineit, Trasler, & Nemer, 1998) and the proximal promoter (+1–[−270]) can be activated by *GATA4* in synergy with WT1 or *NR5A1* (Allali et al., 2011; Miyamoto, Taniguchi, Hamel, Silversides, & Viger, 2008; Tremblay, 1999). We found that *GATA4* + *ZFPM2* co‐transfected with WT1 –KTS isoform consistently activated the human *AMH* proximal promoter around sixfold compared to empty vector controls (Figure 2b). When we tested *GATA4* variants we found control variant p.G221R and p.W228C variant (case1) had a 50% reduction in activity. The other *GATA4* variants show activity comparable to wild‐type (Figure 2b). Similarly, all 10 *ZFPM2* variants showed wild‐type level activation of *AMH* (Figure2b). Taken together, these data suggest that only the *GATA4* p.W228C variant had a loss of activity.

### 2.6 Detection of *ZFPM2*/*GATA4* complex protein interaction

We have found that only *GATA4* variants in the zinc finger domain had a loss of activity in the assays above. As both transactivation assays tested synergy with different co‐factors, we decided to test whether the protein interaction between *GATA4* and *ZFPM2* complex was affected in these variants.

**Figure 3:**
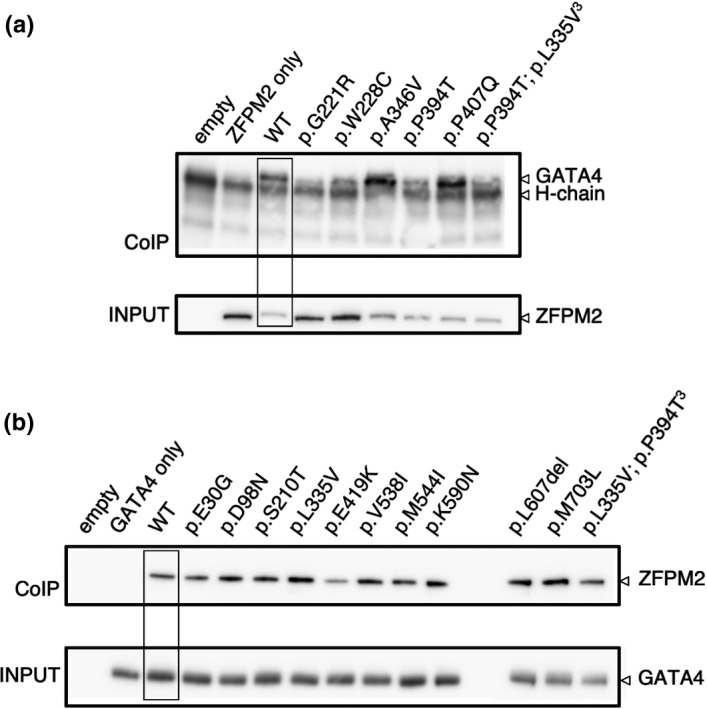
Protein interaction analysis of *GATA4* and *ZFPM2*/*FOG2* complex by co‐immunoprecipitation and Western blot analysis. (a) Detection of *GATA4* variant and wild‐type *ZFPM2* protein complex. Co‐immunoprecipitation was performed by transiently overexpressing wild‐type *ZFPM2* protein with *GATA4* wild‐type or variant protein in HEK293 cells. Pull down of the *GATA4*/*ZFPM2* complex was performed using the FLAG‐tag of the *ZFPM2* protein construct. The complex was then detected by Western blot analysis using the *GATA4* protein antibody. The protein input was verified by Western blotting using *ZFPM2* antibody. An interaction was detected for wild‐type *GATA4* and *ZFPM2* proteins (WT). A decreased interaction was detected for the *GATA4* variants p.G221R (positive control) and p.W228C. (b) Detection of *ZFPM2* variant and wild‐type *GATA4* protein complex. In this case *ZFPM2* wild‐type or variant proteins were over‐expressed with wild‐type *GATA4* protein in HEK293 cells. Pull down of the *GATA4*/*ZFPM2* complex was detected using the HA‐tag of the *GATA4* protein construct. The complex was detected by Western blot analysis using a *ZFPM2* antibody. The protein input was verified by Western blotting using the *GATA4* antibody. An interaction was detected for wild‐type *GATA4* and *ZFPM2* proteins (WT). All *ZFPM2* variants showing the *GATA4*/*ZFPM2* interaction were retained

Individual *GATA4* and *ZFPM2* variant proteins were overexpressed in HEK293 cells and localization and expression was assessed by immunofluorescence and Western blot analysis. All variant proteins were detected at a similar level and pattern to the wild‐type protein (see Figure S1). We then tested the interaction of wild‐type *GATA4*/*ZFPM2* proteins and variant proteins using an in vitro co‐immunoprecipitation assay. Variant protein (eg. *GATA4*) of interest was overexpressed in combination with the wild‐type binding partner (*ZFPM2*) or vice versa in HEK293 cells. The complex was immunoprecipitated using the tag antibody of the wild‐type protein and variant interaction was assessed using the native antibody on Western blot. Wild‐type *GATA4* and *ZFPM2* protein complex was detected (Figure 3). *GATA4* variants p.A394V and p.P407Q interacted with *ZFPM2* at similar levels to the wild‐type protein. Variants p.A346V and double *GATA4*/*ZFPM2* variants (*GATA4*:p.P394T, *ZFPM2*:p.L335V) showed reduced interaction (however the input protein levels were also reduced). Zinc finger variants p.G221R and p.W228C variants were almost undetectable (Figure 3a). We detected a clear band for all *ZFPM2* variants, indicating that their ability to interact with *GATA4* protein in vitro was maintained (Figure 3b).

## 3 DISCUSSION

### 3.1 Re‐curation of *GATA4* and *ZFPM2* variants in 46,XY DSD

Since our initial study, the use of population databases to assess whether a variant is rare enough to cause a condition has advanced significantly. In addition, several studies have highlighted the need to consider various additional genetic factors in DSD such as prevalence, penetrance, genetic and allelic heterogeneity (Whiffin et al., 2017).

The incidence of 46, XY DSD can vary dramatically; from very rare disorders such as complete gonadal dysgenesis affecting 1/20,000 births, to relatively common conditions, such as hypospadias affecting 1/250 male births (Ahmed et al., 2004; Thyen, Lanz, Holterhus, & Hiort, 2006; reviewed in Ohnesorg, Vilain, & Sinclair, 2014). In our study, the majority of patients presented with hypospadias. Given the lack of hormonal data or gonad histology, we considered that this could be isolated hypospadias or represent underlying gonadal dysgenesis. Therefore, we have set our conditional specific maximal allele frequency (AF) threshold to 0.4% (the approximate incidence of hypospadias).

Additionally, around 75% of the cases (case 2/3/5–8/10/14–16) were from distinct ethnic backgrounds such as Pakistan, Cambodia and Indonesia. GnomAD has limited population data for these geographical subsets. Due to the small numbers available from South East Asian countries (like Cambodia and Indonesia), these populations were likely included in the “other” category (Karczewski et al., 2019; Lek et al., 2016). Ancestry based MAF was not used in these cases as low numbers and sample variance of the “other” subset, would be misleading and of limited value. Instead the Popmax filtering AF (95% CI) for each variant is available in GnomAD v2.1 (based on the subpopulation the variant is most commonly observed), was used as an alternative. This feature assists in determining whether a variant is sufficiently rare but does not account for common SNPs in distinct ethnic populations which is only possible with closely matched reference populations (Génin, Letort, & Babron, 2015; MacArthur et al., 2014). However, this first step of variant curation contributed supporting benign evidence when using the maximal incidence for a 46, XY DSD of 0.4% for one out of four *GATA4* variants and six out of ten *ZFPM2* variants which were very close to the threshold (see Table S2).

Obvious gaps in our curation included lack of allelic and segregation data, and genotype–phenotype correlation due to a general lack of clinical information. For these reasons, variant curation relied on only 2–3 scoring criteria, with mainly supporting benign evidence contributing to classification. With the exception of case 1 (p.W228C) which was considered pathogenic. Multiple lines of evidence contributed to pathogenicity including, confirmation of inheritance in an affected family member, genotype–phenotype correlation with a published study (Lourenco et al., 2011), and localized in a well characterized functional domain without benign variation.

A number of DSD genes are known to show variable expressivity and/or incomplete penetrance which complicates interpretation of segregation analysis. Indeed, the first *GATA4* variant described in 46,XY DSD showed variable expressivity in affected males. While incomplete penetrance of CHD was reported for some female relatives who were affected by CHD, while the mother was an unaffected carrier of the variant (Lourenco et al., 2011). Several examples have also been described for *NR5A1*, which plays multiple roles in testis development. Pathogenic variants have been reported to cause a wide range of 46,XY DSD phenotypes, from moderate to severe gonadal dysgenesis, isolated hypospadias, to infertility, (Allali et al., 2011; Robevska et al., 2017; Röpke et al., 2013; Werner et al., 2017), even sometimes within the same family (Eggers et al., 2015). Recently, the phenotypic spectrum of *NR5A1* variants has expanded to include 46,XX individuals presenting with testicular or ovo‐testicular DSD (Knarston et al., 2018; Swartz et al., 2017) or primary ovarian insufficiency (Voican et al., 2013).

### 3.2 Functional analysis substantiates *GATA4* and *ZFPM2* variant classification in 46,XY DSD

Given the complex genetic architecture of 46,XY DSD as described above and the limited patient evidence available to guide curation, we were interested in whether these *GATA4* and *ZFPM2* variants demonstrated aberrant molecular function in the context of testis signaling. Using simplified transactivation assays we tested co‐transcriptional repression or activation by *GATA4* and *ZFPM2* in combination with key co‐factors on gonadal specific promoters. We found that only the *GATA4*:p.W228C variant showed a significant loss of activity (levels similar to the previously identified positive control *GATA4*:p.G221R (Lourenco et al., 2011)), supporting pathogenic classification of this variant. This variant is located in the N‐terminal zinc finger and is thought to be required for interaction with various co‐factors including *ZFPM2* (Crispino et al., 2001; Lourenco et al., 2011; Svensson et al., 1999; Tevosian et al., 2002). Indeed, we found this variant lost protein–protein interaction with known partner *ZFPM2* protein, revealing the likely mechanism underlying its pathogenicity.

These results confirm the outcome of our re‐curation, where the remaining *GATA4* and *ZFPM2* variants had wild‐type activity, providing additional evidence for their benign contribution to DSD. This suggests that these patients should be assessed for other genetic causes. Indeed, three variants in other diagnostic DSD genes (*NR5A1* or *AR*) were found in three of cases presented here (case 4, 15, 16).

Taken together, our study highlights the importance of periodically reassessing DSD gene variants with up to date curation evidence, and disease/tissue‐specific functional assays, particularly when they have been identified as pathogenic in association with another condition. We believe that an integrative approach between the clinical and research setting is essential to further advance our understanding of the genetic basis of DSD.

## 4 MATERIALS AND METHODS

### 4.1 Patient clinical data and DNA

Collaborating clinicians recruited patients for the study, informed consent was obtained and EDTA bloods were collected. Approval for this study was obtained from the Human Ethics Committee of the Faculty of Medicine at the Royal Children's Hospital (application HREC 22073). DNA was extracted by an independent laboratory such as Victorian Clinical Genetics Service (VCGS) or by other hospital providers.

### 4.2 Targeted gene panel, MPS data, and bioinformatics analysis

Targeted MPS gene screening and analysis of these patients has been previously described by Eggers et al. (2016). The bioinformatics pipeline and processing of data is further detailed in Sadedin et al. (2015). All variant annotations were verified in Mutalyzer name checker (https://www.mutalyzer.nl/). Variants of lower quality were verified by Sanger sequencing (details not shown). The sequencing data for each patient is available from the Sequencing Read Archive (SRA) using reference numbers SRP092281 and project PRJNA350857 (Murdoch Children's Research Institute, 2016).

For details regarding initial variant filtering please refer to Eggers et al., 2016. Variants were assessed based on the following criteria: minor allele frequency (MAF) in 1,000 Genomes Project, ESP6,500 and ExAC less than 1%; in silico prediction using (SIFT, Polyphen2, LRT, Mutation Taster); reported or novel based on clinical databases (ClinVar and HGMD); in the case of trio or family analysis inheritance mode was assessed.

### 4.3 Re‐curation of variants using updated databases, tools and guidelines

*GATA4* and *ZFPM2* variants identified in the above study were re‐classified using current population databases GnomAD v2.1 (Karczewski et al., 2019); pathogenicity predictive online tools such as PolyPhen2 (http://genetics.bwh.harvard.edu/pph2) (Adzhubei et al., 2010) Mutation taster (http://www.mutationtaster.org/) (Schwarz, Rödelsperger, Schuelke, & Seelow, 2010) and SIFT (https://sift.bii.a-star.edu.sg/) (Sim et al., 2012). We considered MAF and Popmax Filtering AF (95% CI) to be less than 0.4% (incidence of hypospadias) as sufficiently rare to cause the condition. See Table S2 for more details on curation evidence and scoring criteria used for variant classification for each patient presented in this study.

### 4.4 Variant expression constructs

The variant overexpression vectors for *ZFPM2* (NM_012082.3: c.89A > G; c.1612G > A, c.1255G > A, c.292G > A, c.292G > A, c.629G > C, c.1003C > G, c.1632G > A, c.1770G > C, c.1816_1818del, c.2107A > C) and *GATA4* (NM_002052.4: c.684G > C, c.1037C > T, c.1180C > A, c.1220C > A) were created by site‐directed mutagenesis (QuickChange II XL Site‐directed Mutagenesis Kit; Agilent Technologies Inc.) according to the manufacturer's instructions (see Table S3 for specified primer sequences). Mutagenesis was performed using the mammalian expression vector containing the human cDNA ORF for: pCMV6‐Entry‐*hGATA4* (RC210945 [NM_002052.4]; from OriGene Technologies Inc.) and pCMV6‐Entry‐*hZFPM2* (RC214338 [NM_012082.3]; from OriGene Technologies Inc.) both with a C‐terminal Myc‐DDK tag. Prior to introducing mutations to the pCMV6‐Entry‐*hGATA4* the vector was modified to remove the Myc‐DDK tag and introduce a C‐terminal HA tag. Sanger sequencing using universal vector primers was used to confirm the introduction of the correct variant.

### 4.5 Transactivation assays

Luciferase transactivation assays for mouse enhancer of Tesco (pGL4‐*mTesco*) and human proximal promoter of *AMH* (pGL4‐*hAMH*, +10–[−270]bp, cloned in‐ house) were setup using HEK293‐T human embryonic kidney cells and Lipofectamine‐2000 as the transfection reagent. Cells were setup in 96‐well plates, co‐transfected with promoter vector (75 ng pGL4‐mTesco or 100 ng pGL4‐hAMH) and 5 ng of *Renilla* (pRL‐TK) as a marker of transfection efficiency. For the mTesco assay: transcription factors were also transfected with or without 40 ng of wild‐type pCMV6‐Entry‐hNR5A1 (RC207577; OriGene Technologies Inc.); 40 ng of pCMV6‐*GATA4* wild‐type (RC210945, OriGene Technologies Inc.) or mutant vectors; and 40 ng pCMV6‐*ZFPM2* wild‐type (RC214338; OriGene Technologies Inc.) or mutant vector. Total DNA for each mTesco assay was 200 ng, pCMV‐empty was used to adjust the total DNA for each well. In the case of the hAMH assay: 45 ng wild‐type pcDNA‐WT1 KTS−/−; with or without) 15 ng pCMV‐*GATA4* (wild‐type or mutant); and 15 ng pCMV‐*ZFPM2* (wild‐type or mutant), were used at a respective ratio of 3:1:1 (WT1:*GATA4*:*ZFPM2*). Total DNA for each hAMH assay was 180 ng, pCMV‐empty was used to adjust the total DNA for each well. Assays were lysed 24 hr post‐transfection and luciferase activity was measured using the dual‐luciferase reporter assay (Dual‐Luciferase Reporter 1,000 Assay System Kit; Promega) on an Infinite M200 Pro plate reader (Tecan). Each data point represents the average ratio of firefly to renilla luciferase for each condition (performed in triplicate), normalized to the empty vector control (fold change relative to the negative control). The standard error of the mean is shown for three to four independent experiments that were run in technical triplicate.

### 4.6 Protein overexpression analysis (immunofluorescence and Western blot)

Protein for immunofluorescent imaging was prepared by seeding HEK293‐T cells on 8‐well chamber slides, 200 ng of individual overexpression constructs was transfected with Lipofectamine 2000 (Invitrogen). After 24 hr post‐transfection cells were processed for staining by removing media, briefly washing cells with ice‐cold PBS, fixing cells for 10 min with 4% PFA, 10 min permeabilization with 1% triton‐X‐100 in PBS, and blocking with 2% BSA in PBS. Cells were then incubated overnight with primary antibody in 1% BSA PBS as follows: for *GATA4* (wild‐type or mutant) over expression, rabbit polyclonal anti‐*GATA4* antibody at 1:200 (Abcam, ab84593); and for *ZFPM2* (wild‐type or mutant) over expression, mouse monoclonal anti‐*FOG2* (H5) at 1:1000 (Santa Cruz, sc398011). The next day, cells were washed several times with PBS before incubating with the following secondary antibodies in 1% BSA in PBS: for *GATA4* overexpression staining secondary antibodies donkey anti‐rabbit Alexa‐488 (1:1,000; green, Invitrogen) and for *ZFPM2* overexpression donkey anti‐mouse Alexa‐488 (1:1,000; green, Invitrogen). Nuclear counterstaining with DAPI (blue) was also performed. Images were acquired on a Zeiss AXIO Imager M1 for each overexpressed gene the variants were captured using the same settings as for the wild‐type image.

Protein analysis using Western blot was prepared by seeding 4 × 10^5^ HEK293‐T cells in a 24‐well plate and transfecting 800 ng pCMV6‐Entry‐*GATA4* (NM_002052.4) (RC210945, OriGene Technologies Inc.) or pCMV6‐Entry‐*ZFPM2* (NM_012082.3) overexpression construct (RC214338; OriGene Technologies Inc.) (wild‐type or mutant) with Lipofectamine 2000 (Invitrogen). Protein was harvested 24 hr post‐transfection by removing media and washing the cells with ice‐cold PBS, and lysed using Pierce IP lysis buffer (25 mM Tris HCl pH7.4, 150 mM NaCl, 1% NP‐40, 1 mM EDTA, 5% glycerol) with protease inhibitors (COmplete ULTRA tablets, EDTA free EASYpack, Roche, 05892791001). Protein was quantified using the Pierce BCA protein assay kit (ThermoScientific, 23227), along with BSA protein standards. Five micrograms of total protein was run on a 10% Bis‐Tris gel with MOPS buffer (*GATA4*), or 4%–12% Bis‐Tris with MOPS buffer (Invitrogen) (*ZFPM2*), transferred to PVDF membrane, blocked using 5% skim milk powder/TBST and incubated with rabbit polyclonal anti‐*GATA4* antibody (1:2000, Abcam, ab84593); or mouse monoclonal anti‐*FOG2* (H5) (1:2000, Santa Cruz, sc398011) overnight at 4°C. After washing with TBST, swine anti‐rabbit HRP (1:10000 DAKO P0399) was incubated at room temperature for 2 hr. After blot washing the Amersham ECL Prime Western blotting detection reagent was used and visualized with the GE Healthcare Life Sciences ImageQuant LAS 4000. Blots were washed and then incubated with loading control—anti‐alpha Tubulin (HRP) (1:5000, Abcam, ab40742) or anti‐GAPDH (HRP) (1:5000, Abcam, ab9482), washed and detected as mentioned above.

### 4.7 Protein interaction detected by co‐immunoprecipitation and Western blotting

Interaction of *GATA4* and *ZFPM2* proteins was detected as follows: HEK293‐T cells were seeded at 1.6 × 10^6^ in 6‐well plates, transfected with 1.5 ug of pCMV6‐GATA (HA‐tag) (modified pCMV6‐*GATA4*, RC210945, OriGene Technologies Inc.) containing the human cDNA ORF of *GATA4* (NM_002052.4) (wild‐type or variant); and 1.5 ug pCMV6‐Entry‐hZFPM2 (myc‐DDK tag) (RC214338; OriGene Technologies Inc.) containing the human cDNA ORF of *ZFPM2* (NM_012082.3) (wild‐type or variant) with Lipofectamine 2000. After 24 hr, cells were washed and harvested in 500 ul of Pierce IP lysis solution with protease inhibitors (COmplete ULTRA tablets, EDTA free EASYpack, Roche, 05892791001), protein lysates were quantitated using the Pierce protein assay kit. For each IP reaction 500 ug of protein lysate (in a total volume 250 ul) was incubated with 1.5 ul rabbit anti‐HA (Sigma, H6908) (when testing for *ZFPM2* protein interaction) or 1.5 ul rabbit anti‐FLAG (Sigma, F7425) (when testing for *GATA4* protein interaction) overnight on a suspension mixer with inversion at 4°C degrees. The following day, 20 ul A/G plus agarose beads (Santa Cruz, sc2003) was directly added to each IP reaction and incubated for 4 hr on a suspension mixer at 4°C degrees. The IP reaction was then centrifuged at 587g for 5 min at 4°C degrees, supernatant carefully removed and the pellet washed 4–5 times with 500 ul PBS, in the same manner as mentioned above. The IP protein was then resuspended directly in 40 ul 2x SDS protein loading buffer (100 mM Tris‐Cl [pH 6.8], 4% [w/v] SDS, 0.2% [w/v] bromophenol blue, 200 mM DTT), boiled at 95–100°C for 5 min to denature the protein and release it from the beads. Western blot analysis of the co‐immunoprecipitation was setup as described above: for each IP reaction 15 ul was run per lane; while the 5% of the input sample was run on a separate gel as a control. When testing for *GATA4* mutant protein interactions, anti‐*GATA4* was used to probe the IP reactions and anti‐*ZFPM2* was used to probe the input (as mentioned above). When testing for *ZFPM2* mutant protein interactions, anti‐*ZFPM2* was used to probe the IP reactions and anti‐*GATA4* was used to probe the input (as mentioned above).

## CONFLICT OF INTEREST

Dr Davis reports having received honoraria from Besins Healthcare and Pfizer Australia and has been a consultant to Mayne Pharmaceuticals, Lawley Pharmaceuticals and Que Oncology. All other authors declare no conflict of interest.

## AUTHOR CONTRIBUTIONS

The manuscript was compiled and written by J.vdB, K.A and A.S. Patient database, sample preparation, sequencing and analysis was performed by J.vdB, G.R, S.E and K.A. In vitro and functional studies were performed by J.vdB, while supervision and project guidance were provided by K.A and A.S. Management and recruitment of patients involved in the study was coordinated by S.F, S.R, S.G, P.B, C.K, S.K, A.J, C.S, J.R, I.A, S.D, M.A, and V.H. All authors were involved in critical review of the manuscript.

## Data Availability

The data that support the findings of this study are openly available in Sequence Read Archive (SRA) at https://www.ncbi.nlm.nih.gov/sra/PRJNA350857, reference number [SRP092281].
