# Supplementary material for: Analysis of variants in GATA4 and FOG2/ZFPM2 demonstrates benign contribution to 46,XY disorders of sex development
Source: Mol Genet Genomic Med. 2020 Jan 21;8(3):e1095. doi: 10.1002/mgg3.1095 (PMC7057099; doi:10.1002/mgg3.1095)
Supplement: Supplementary file 1 [file MGG3-8-e1095-s001.pdf]

**Supplementary Table S1. Other variants identified in DSD genes for patients**

| Patient id | Other DSD gene | Zygosity | HGVS transcript | HGVS protein | ExAC | Poly phen2 | Mutation Taster | SIF T | Grantham score | ClinVar: Condition | chr. coordinates (hg19) |
|---|---|---|---|---|---|---|---|---|---|---|---|
| **4** | *AR* | X-linked | NM_000044.4: c.2599G>A | NP_000035.2: p.V867M | not found | B | D | T | 21 | Pathogenic (AIS; MIM# 300068) | chrX:66942818 (rs137852564) |
| **15** | *AR* | X-linked | NM_000044.4: c.2191G>A | NP_000035.2: p.V731M | 0.000047 | B | D | D | 21 | Prostate cancer, somatic (Newmark et al. 1992) | chrX: 66937337 (rs137852571) |
| **16** | *NR5A1* | het | NM_004959: c.G251A | NP_004950.2: p.R84H | 0.000008 | D | D | D | 84 | 46,XY DSD (Robevska et al. 2017) | chr9:127262988 |

Three 46,XY DSD patients in this study had an additional variant identified in a known DSD gene.
The AR variant in case 4 and the NR5A1 variant in case 16 have been previously reported in association with 46,XY DSD.

**Supplementary Table S2.** Evidence for curaion of GATA4 variants

| | | | | | | | | |
|---|---|---|---|---|---|---|---|---|
| **Gene** | | ***GATA4: NM_002052.4*** | | | | | | |
| **OMIM phenotype (MIM#; inheritance)** | | *Testicular anomalies with or without congenital heart disease (TACHD; MIM# 615542; AD)*<br>*Atrial septal defect 2 (ASD2; MIM# 607941; AD)*<br>*Atrioventricular septal defect 4 (AVSD4; MIM# 614430; AD)*<br>*Tetralogy of Fallot (TOF; MIM# 187500; AD)*<br>*Ventricular septal defect 1 (VSD1; MIM# 614429; AD)* | | | | | | |
| **Variant** | **NM_002052.4** | c.684G>C | *c.1037C>T* | c.1180C>A | | c.1220C>A | | |
| | **NP_002043** | p.W228C | *p.A346V* | p.P394T | | p.P407Q | | |
| **Genomic annotation (hg19)** | | chr8:11606495-11606496 | *chr8:11614483-11614484* | chr8:11615835-11615836 | | chr8:11615875-11615876 | | |
| **Variant based evidence:** | | | | | | | | |
| **Population database:** | **GnomAD (overall or specificed)** | Absent | 0.00152 | 0.002027 | | 0.0004667 | | |
| | **Popmax filtering AF** | n/a | 0.002475 (EUR) | 0.01716 (SA) | | 0.002845 (EA) | | |
| | **Homozygous observations** | none | none | 9 | | none | | |
| | **Score** | **PM** | **PP** | **BS** | | **PP** | | |
| **Polyphen2/Mutation Taster/SIFT** | | D/D/D | B/ B/T | B/ B/T | | B/D/D | | |
| **Conservation (phyloP score)** | | High (6.0) | moderate (1.95) | low (-0.377) | | moderate (2.02) | | |
| **Grantham score** | | 215 | 64 | 38 | | 75 | | |
| **Computational and predictive data score** | | **PP** | **BP** | **BP** | | **BP** | | |
| **Functional Data** | | Well studied mouse model within functional domain showing a deleterious effect (Tevosian, 2002). Other pathogenic variants identified within functional domain (Lourenco, 2011) without bening function. **PM** | n/a | n/a | | n/a | | |
| **Variant reported in alternative database (ClinVar)** | | none | AVSD4; MIM# 614430 **Conflicting** reports of pathogenicity | AVSD4; MIM# 614430 Benign **BP** | | (TOF; MIM#187500);(AVSD4; MIM# 614430); (ASD2; MIM# 607941); AVSD4; MIM# 614430) **Conflicting** reports of pathogenicity | | |
| **Patient guided evidence** | | 1 | 2 | 3 | 4 | 5 | 6 | 7 |
| **Ancestry** | | European | Cambodian | Pakistan | European | Indonesian | Indonesian | Indonesian |
| **Ancestry based MAF** | | not found | 0.0008306 (other) | 0.01842 (SA) | not found | 0.0002767 (other) | | |
| **Zygosity** | | het | het | het | het | het | het | het |
| **Segregation data** | | Maternally inherited. Co-segregation with phenotype in other affected family member. **PP** | n/a | n/a | n/a | n/a | n/a | n/a |
| **Inheritance data** | | n/a | n/a | n/a | n/a | n/a | n/a | n/a |
| **Alternative causative variant** | | none | none | ZFPM2: NM_012082.3: c.1003C>G:p.L335V | AR: NM_000044.4: c.2599G>A; NP_000035.2:p.V867M (phenotype match) **BP** | none | none | none |
| **Other evidence** | | | | | | complex phenotype not in line with genotype: phenotype for variants in this gene. **BP** | | |
| **Criteria Summary** | | 2PM+2PP | PP+BP+C | BS+2BP | BS+3BP | PP+2BP+C | PP+BP+C | PP+BP+C |
| **Conclusion** | | Pathogenic | Likely Benign | Benign | Benign | Benign | Likely Benign | Likely Benign |

**Supplementary Table S2.** Evidence for curaion of ZFPM2/FOG2 variants

| | | | | | | | | | | | | |
|---|---|---|---|---|---|---|---|---|---|---|---|---|
| **Gene** | ***ZFPM2 (FOG2): NM_012082.3*** | | | | | | | | | | | |
| **OMIM phenotype (MIM#, inheritance)** | *46XY sex reversal 9 (SRXY9; MIM# 616067; AD)*<br>*Diaphragmatic hernia 3 (DIH3; MIM# 610187)*<br>*Tetralogy of Fallot (TOF; MIM# 187500; AD)* | | | | | | | | | | | |
| **Variant** **NM_012082.3** | c.89A>G | c.1612G>A | c.89A>G | *c.1255G>A* | c.292G>A | | c.629G>C | c.1003C>G | c.1632G>A | c.1770G>C | c.1818_1820del | c.2107A>C |
| **NP_036214.2** | p.E30G | p.V538I | p.E30G | *p.E419K* | p.D98N | | p.S210T | p.L335V | p.M544I | p.K590N | p.L607del | p.M703L |
| **Genomic annotation (hg19)** | *chr8:106431420-106431421* | *chr8:106813922-106813923* | *chr8:106431420-106431421* | *chr8:106813565-106813566* | *chr8:106456600-106456601* | | *chr8:106801042-106801043* | *chr8:106813313-106813314 (rs376235097)* | *chr8:106813942-106813943 (rs187043152)* | *chr8:106814080-106814081* | *chr8:106814125-106814127* | *chr8:106814417-106814418 (rs121908603)* |
| **Variant based evidence:** | | | | | | | | | | | | |
| **Population database** **GnomAD (overall)** | 0.002691 | 0.00022 | 0.002691 | 0.00002411 (allele count 4) | 0.002628 | | 0.002235 | 0.0005956 | 0.00334 | 0.000004014 (allele count 1) | 0.000004018 (allele count 1) | 0.0004784 |
| **Popmax filtering AF** | 0.004302 (EUR) | 0.0009866 (SA) | 0.004302 (EUR) | 0.00000961 (LAT) | 0.004543 (EUR) | | 0.004076 (LAT) | 0.00478 (SA) | 0.004531 (EUR) | n/a | n/a | 0.00572 (EA) |
| **Homozygous observations** | 5 | none | 4 | none | 5 | | 1 | 1 | 4 | none | none | none |
| **Score** | **BS** | **PP** | **BS** | **PP** | **BS** | | **BS** | **BS** | **BS** | **PP** | **PP** | **BS** |
| **Polyphen2/Mutation Taster/SIFT** | PD/D/D | PD/D/D | PD/D/D | B/D/T | B/D/D | | B/D/T | PD/D/D | B/D/T | PD/D/D | D/D/T | D/D/T |
| **Conservation (PhyloP score)** | Moderate (4.64) | Moderate (4.46) | Moderate (4.64) | Moderate (3.46) | Moderate (3.91) | | Moderate (2.78) | Moderate (2.86) | Moderate (4.48) | Moderate (1.77) | n/a | High( 5.12) |
| **Grantham score** | 98 | 29 | 98 | 56 | 75 | | 58 | 32 | 10 | 94 | N/A | 15 |
| **Computational and predictive data score** | **PP** | **BS** | **PP** | **BS** | **BS** | | **BS** | **BS** | **BS** | **PP** | **PP** | **PP** |
| **Functional Data** | n/a | n/a | n/a | n/a | n/a | | n/a | n/a | n/a | n/a | Variant within the 6th Zinc finger CCHC FOG-type 3 | n/a |
| **Variant reported in alternative database (ClinVar)** | (SRXY9; MIM# 616067); (TOF; MIM# 187500) Likely benign - **BP** | none | (SRXY9; MIM# 616067); (TOF; MIM# 187500) Likely benign - **BP** | none | (SRXY9; MIM# 616067) Benign and VUS -**BP** | | (SRXY9; MIM# 616067) Benign - **BP** | none | (SRXY9; MIM# 616067 (Bashamboo, 2014)); (TOF; MIM# 187500 (Luca, 2011)); - Conflicting interpretation - **C** | none | none | (DIH3; MIM# 610187); (TOF; MIM# 187500) - Pathogenic |
| **Patient guided evidence** | **8** | | **9** | | **10** | **11** | **12** | **3*** | **13** | **14** | **15** | **16** |
| **Ancestry** | Pakistan | | Eastern European | | Indonesian | European | European | Pakistan | European | Pakistan | Cambodian | Indonesian |
| **Ancestry based MAF** | 0.001243 (SA) | 0.001307 (SA) | 0.004557 (EUR) | 0.00002662 (EUR) | 0.003119 (other) | 0.004543 (EUR) | 0.002882 (EUR) | 0.005133 (SA) | 0.005248 (EUR) | not found | not found | not found |
| **Zygosity** | het | het | het | het | het | het | het | het | het | het | het | het |
| **Segregation data** | n/a | n/a | n/a | n/a | n/a | n/a | n/a | n/a | n/a | n/a | n/a | n/a |
| **Inheritance data** | n/a | n/a | n/a | n/a | n/a | n/a | maternal | n/a | n/a | n/a | not maternal (paternal unknown) | n/a |
| **Alternative causative evidence** | | | | | | | | GATA4: NM_002052.4:c.1180 C>A:p.P394T - Benign | n/a | n/a | AR:NM_000044.4:c.2 191G>A:p.V731M (phenotype match) - **BP** | NR5A1:NM_004959:c. 251G>A:p.R84H (phenotype match), Robevska (2017) - **BP** |
| **Other evidence** | | | | | | | | | Reported homozygous in SRXY; MIM# 616067 (Bashamboo, 2014) | | | |
| **Criteria Summary** | BS+PP+BP | PP+BS | BS+PP+BP | PP+BS | 2BS+BP | 2BS+BP | 2BS+BP | 2BS | 2BS+C | 2PP | 2PP+BP | BS+PP+BP |
| **Conclusion** | Likely benign | VUS | Likely benign | VUS | Benign | Benign | Benign | Benign | Likely Benign | VUS | VUS | Benign |

**Summary of evidence used for variant re-curation**

**1. Population frequency:**

MAF: Popmax filtering AF used

Threshold used: incidence of hypospadias 1/250 (0.4%).

Scoring criteria: Absent from population databases - PM; <0.4% incidence of hypospadias - PP; >0.4% incidence of hypospadias - BS

**2. Protein prediction informatics/conservation:**

Tools used:

SIFT predictions from https://sift.bii.a-star.edu.sg/

Polyphen2 predictions are from http://genetics.bwh.harvard.edu/pph2/.

Mutation Taster predictions are from http://www.mutationtaster.org/.

Conservation score was determined by manual inspection of the PhyloP conservation tracks in UCSC (https://genome.ucsc.edu/). A score >4.88 (pink hat) is considered high, a score between 0 and 4.88 (blue) is considered moderate.

Grantham score: Based on a given amino acid substitution the algorithm takes into account chemical composition and physicochemical similarity between amino acid changes, the change is given a score (0-215): <65 amino acid change similar/tolerated; 65-100 moderate amino acid change; >100 likely deleterious amino acid change.

Scoring criteria: consistent damaging predictions and high conservation - PP; inconsistent predictions - BS

**3. Variant previously reported (ClinVar):**

consistent benign annotation - BP; consistent pathogenic annotation - PP; conflicting interpretation - C

**4. Alternative causative evidence:**

Previously identified variant in a known DSD gene, where genotype:phenotype consistent - BP

Light grey highlights criteria used for variant scoring.

Conclusion: Variant classification based on rules for combining scoring criteria (Richards et al. 2015).

Evidence key: BS - strong benign; BP - supporting benign; PS - strong pathogenicity; PM - moderate pathogenicity; PP - supporting pathogenicity

**Supplementary Figure S1. Localisation and detection of overexpressed GATA4 and ZFPM2/FOG2 protein variants**

**A.** GATA4 wild-type and variant overexpression in HEK293 cells, detected by immunofluorescence

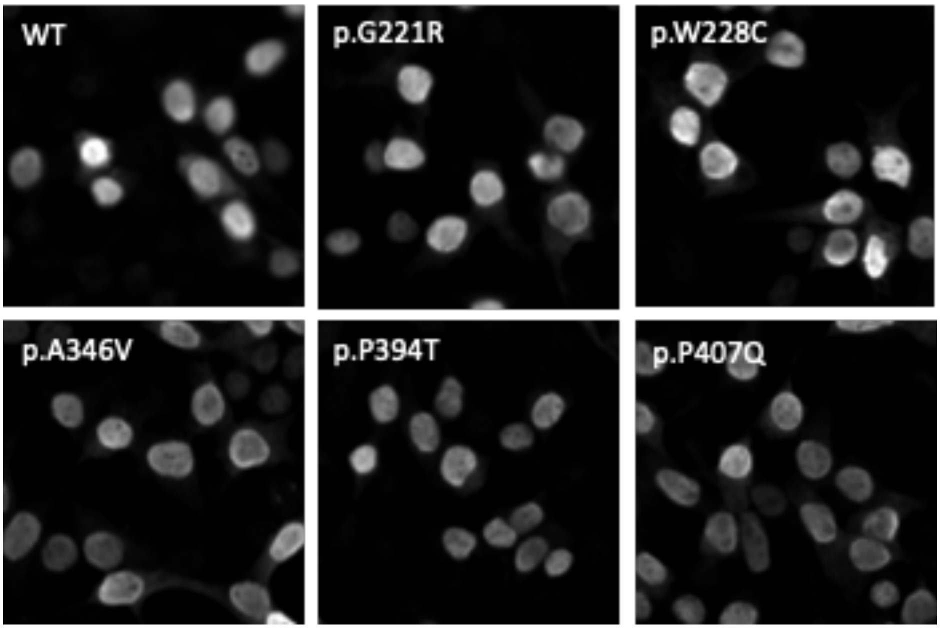


**B.** ZFPM2/FOG2 wild-type and variant overexpression in HEK293 cells, detected by immunofluorescence.

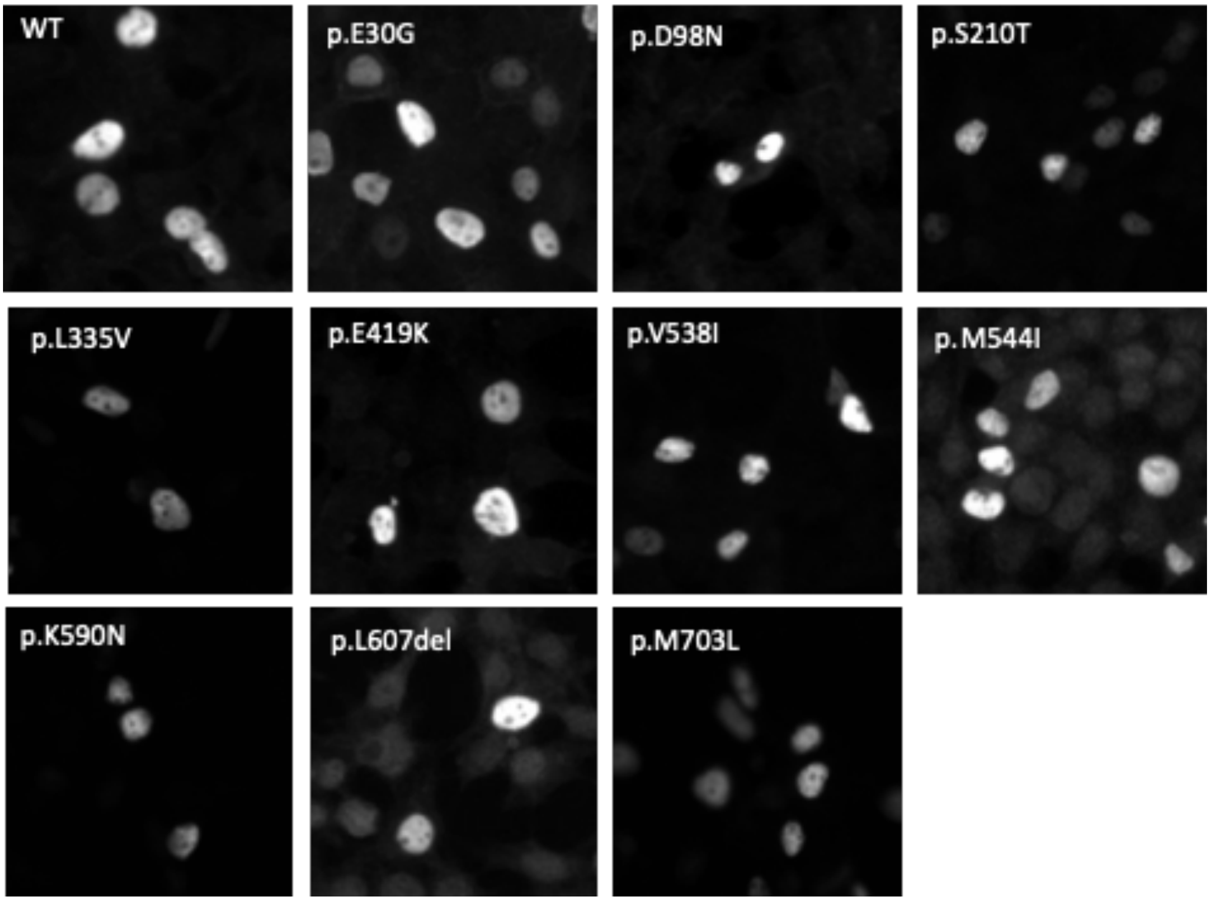

**C.** Overexpression of GATA4 wild-type and variants in HEK293 cells, detected by Western blotting

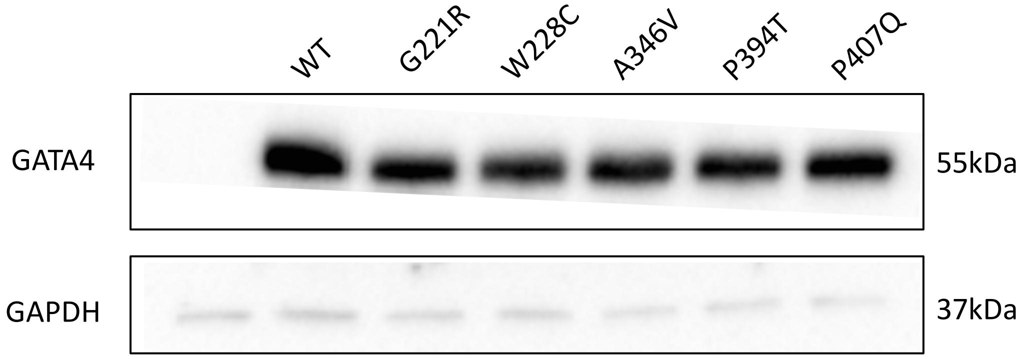


**D.** Overexpression of ZFPM2 wild-type and variants in HEK293 cells, detected by Western blotting

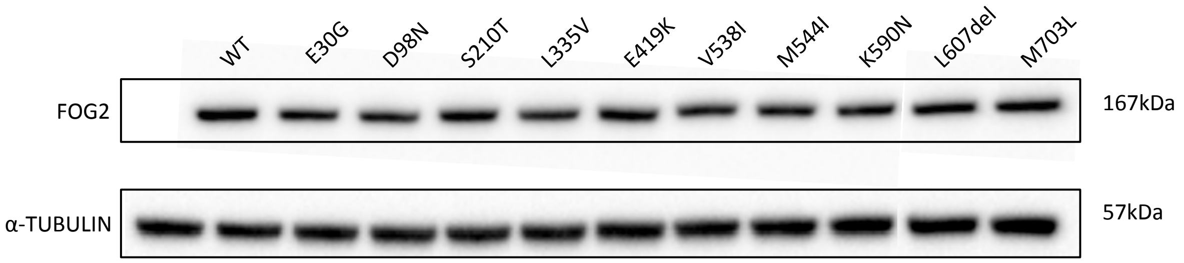


**Supplementary Figure 1.**

**A and B.** Overexpression of GATA4 (A) or ZFPM2 (B) wild-type (WT) or variant constructs in HEK293 cells. Cells were processed for immunofluorescence using primary antibody for GATA4 (A) or ZFPM2 (B), cells were counterstained with DAPI to confirm nuclear localization of overexpressed protein similar to the WT protein (DAPI staining not shown). Images were taken at X20 magnification with same settings as wild-type control.
**C and D.** Overexpression of GATA4 (C) or ZFPM2 (D) wild-type (WT) or variant constructs in HEK293 cells. Cells were harvested for protein analysis and detected using Western blotting. Overexpressed variant proteins were detected with primary GATA4 (C) or FOG2 (D) antibody and GAPDH (C) or alpa-tubulin (D) antibodies were used as a loading control. Western blot analysis showed GATA4 and ZFPM2 variant proteins were expressed at similar levels to wild-type protein.

**Supplementary Table S3. Mutagenesis primers**

QuikChange mutagenesis primers designed using the Agilent software
https://www.agilent.com/store/primerDesignProgram.jsp

| Name | Tm | Base count | Sequence (5'-3') |
|---|---|---|---|
| hZFPM2_c.A89Gp.E30G_for | 79.5 | 39 | ggagatgatgtctgtttcccctgatggacattcttcttc |
| hZFPM2_c.A89Gp.E30G_rev | 79.5 | 39 | gaagaagaatgtccatcaggggaaacagacatcatctcc |
| hZFPM2_c.G292Ap.D98N_for | 80.31 | 31 | gctctcctggtccattccagtcgtctgtctc |
| hZFPM2_c.G292Ap.D98N_rev | 80.31 | 31 | gagacagacgactggaatggaccaggagagc |
| hZFPM2_c.G629Cp.S210T_S | 79.14 | 33 | aaggctacaagctgccactcagatgactctcac |
| hZFPM2_c.G629Cp.S210T_AS | 79.14 | 33 | gtgagagtcatctgagtggcagcttgtagcctt |
| hZFPM2_c.C1003Gp.L335V_S | 78.98 | 31 | ccccctggtgctagtgtaaaatgcaccgtct |
| hZFPM2_c.C1003Gp.L335V_AS | 78.98 | 31 | agacggtgcattttacactagcaccaggggg |
| hZFPM2_c.G1255Ap.E419K_for | 78.1 | 35 | ttggctctggggaagtttgcttctggtcaataagt |
| hZFPM2_c.G1255Ap.E419K_rev | 78.1 | 35 | acttattgaccagaagcaaacttccccagagccaa |
| hZFPM2_c.G1612Ap.V538I_S | 79.27 | 35 | gtagtagctaccctcccatcatttacagccctttg |
| hZFPM2_c.G1612Ap.V538I_AS | 79.27 | 35 | caaagggctgtaaatgatgggagggtagctactac |
| hZFPM2_c.G1632Ap.M544I_for | 79.1 | 33 | gtagccccccttgggtatcaaagggctgtaaatg |
| hZFPM2_c.G1632Ap.M544I_rev | 79.1 | 33 | catttacagccctttgatacccaagggggctac |
| hZFPM2_c.G1770Cp.K590N_S | 78.1 | 35 | agttccctagtgtgtcagaaaacatgcctgaagct |
| hZFPM2_c.G1770Cp.K590N_AS | 78.1 | 35 | agcttcaggcatgttttctgacacactagggaact |
| hZFPM2_c.1816_1818del_for | 79 | 32 | ccaaacctccataaacctcaacccagctgctc |
| hZFPM2_c.1816_1818del_rev | 79 | 32 | gagcagctgggttgaggtttatggaggtttgg |
| hZFPM2_c.A2107C p.M703L_S | 79.14 | 33 | gccggcacgaaacatacctggtccacaaacagt |
| hZFPM2_c.A2107C p.M703L_AS | 79.14 | 33 | actgtttgtggaccaggtatgtttcgtgccggc |
| hGATA4_c.G661A:p.G221R_S | 79.14 | 33 | cagagagtgtgtcaactgtagggctatgtccac |
| hGATA4_c.G661A:p.G221R_AS | 79.14 | 33 | gtggacatagccctacagttgacacactctctg |
| hGATA4_c.G684C:p.W228C_S | 80.02 | 25 | caccccgctctgcaggcgagatggg |
| hGATA4_c.G684C:p.W228C_AS | 80.02 | 25 | cccatctcgcctgcagagcggggtg |
| hGATA4_c.C1037T:p.A346V_S | 80.13 | 27 | cccgccagcggtgtttccagcaactcc |
| hGATA4_c.C1037T:p.A346V_AS | 80.13 | 27 | ggagttgctggaaacaccgctggcggg |
| hGATA4_c.C1180A:p.P394T_S | 80.13 | 27 | gtctggccatgggacctccatccaccc |
| hGATA4_c.C1180A:p.P394T_AS | 80.13 | 27 | gggtggatggaggtcccatggccagac |
| hGATA4_c.C1220A:p.P407Q_S | 80.31 | 31 | ccctgaagctctcccaacaaggctatgcgtc |
| hGATA4_c.C1220A:p.P407Q_AS | 80.31 | 31 | gacgcatagccttgttgggagagcttcaggg |

Newmark JR, Hardy DO, Tonb DC, Carter BS, Epstein JI, Isaacs WB, Brown TR, Barrack ER. 1992. Androgen receptor gene mutations in human prostate cancer. Proceedings of the National Academy of Sciences 89: 6319–6323.

Robevska G, van den Bergen JA, Ohnesorg T, Eggers S, Hanna C, Hersmus R, Thompson EM, Baxendale A, Verge CF, Lafferty AR, Marzuki NS, Santosa A, et al. 2017. Functional characterization of novel NR5A1 variants reveals multiple complex roles in disorders of sex development. Hum. Mutat. 22: 125–139.
